# Citation advantage for open access articles in European Radiology

**DOI:** 10.1007/s00330-019-06389-0

**Published:** 2019-08-19

**Authors:** Rayan H. M. Alkhawtani, Thomas C. Kwee, Robert M. Kwee

**Affiliations:** 1 Department of Radiology, Nuclear Medicine and Molecular Imaging, University Medical Center Groningen, University of Groningen, Hanzeplein 1, P.O. Box 30.001, 9700 RB Groningen, the Netherlands; 2 Department of Radiology, Zuyderland Medical Center, Heerlen/Sittard/Geleen, The Netherlands

**Keywords:** Bibliometrics, Open access publishing, Radiology

## Abstract

**Objective:**

To investigate whether there is a difference in citation rate between open access and subscription access articles in the field of radiology.

**Methods:**

This study included consecutive original articles published online in *European Radiology*. Pearson *χ*^2^, Fisher’s exact, and Mann-Whitney *U* tests were used to assess for any differences between open access and subscription access articles. Linear regression analysis was performed to determine the association between open access publishing and citation rate, adjusted for continent of origin, subspeciality, study findings in article title, number of authors, number of references, length of the article, and number of days the article has been online. In a secondary analysis, we determined the association between open access and number of downloads and shares.

**Results:**

A total of 500 original studies, of which 86 (17.2%) were open access and 414 (82.8%) were subscription access articles, were included. Articles from Europe or North America were significantly more frequently published open access (*p* = 0.024 and *p* = 0.001), while articles with corresponding authors from Asia were significantly less frequently published open access (*p* < 0.001). In adjusted linear regression analysis, open access articles were significantly more frequently cited (beta coefficient = 3.588, 95% confidence interval [CI] 0.668 to 6.508, *p* = 0.016), downloaded (beta coefficient = 759.801, 95% CI 630.917 to 888.685, *p* < 0.001), and shared (beta coefficient = 0.748, 95% CI 0.124 to 1.372, *p* = 0.019) than subscription access articles (beta coefficient = 3.94, 95% confidence interval 1.44 to 6.44, *p* = 0.002).

**Conclusion:**

Open access publishing is independently associated with an increased citation, download, and share rate in the field of radiology.

**Key Points:**

• *A minority of articles are currently published open access in European Radiology.*

• *European and North American authors tend to publish more open access articles than Asian authors.*

• *Open access publishing seems to offer an independent advantage in terms of citation, download, and share rate.*

## Introduction

A citation is the acknowledgment that a scientific article has been referenced by another article [1]. The number of citations a published article receives can be considered a measure of its impact in the scientific community [1]. The citation rate directly influences scientometric indicators such as the h-index (an indicator of an author’s research performance [2]) and impact factor (an indicator of a journal’s prestige [3]). Open access refers to the practice of making research outputs such as scientific articles freely available online to all users. A possible benefit is that open access articles may be viewed and cited more frequently than articles that are only available to subscribers. However, open access publishing comes at a price: current prices for open access options in radiology journals range from $750 to $4,000 (median $3,000) [4]. Furthermore, there is currently no unambiguous evidence to support the hypothesis that open access articles are cited more frequently. A randomized prospective trial in physiology journals found no difference in citation rates between open access and subscription access articles in the first year after publication [5]. Studies in the field of oncology [6] and dentistry [7] found conflicting results. A recent study showed a higher citation rate for open access journals in general, but the effect was not uniform across different types of journals [8]. A recent study among radiology journals found no statistically significant differences in journal impact measures (including number of journal citations and impact factors) between journals with and without open access options [4]. To our knowledge, no such research has been performed yet for individual radiological articles. Therefore, the primary objective of our study was to investigate whether there is a difference in citation rate between open access articles and subscription access articles in the field of radiology. In order to measure the potential broader impact of open access publishing, the secondary objective was to investigate its effect with regard to download and share rate.

## Methods

Ethics committee approval was not applicable for this literature study.

### Data collection

A research fellow (R.H.M.A.) included all consecutive original articles published online in *European Radiology* between April 23, 2015, and July 6, 2017. Review articles (including guidelines, consensus developments, narrative reviews, and systematic reviews/meta-analyses), editorials, letters to the editor, and case reports were excluded. The following data were extracted for each included article: whether or not the article was published open access, continent of origin of the corresponding author, subspeciality (breast, cardiac, computed tomography, computer applications, contrast media, education, emergency, experimental, forensic medicine, gastrointestinal-abdominal, head-neck, health economy, magnetic resonance, molecular imaging, musculoskeletal, neuroradiology, nuclear medicine, oncology, pediatric, physics, ultrasound, urogenital, or vascular-interventional), mentioning of study findings in the article title, number of authors, number of references, length of the article in pages, number of days the article has been online (calculated as the number of days between the date the article was analyzed for this study and the date the article was published online on the *European Radiology* website, https://link.springer.com/journal/volumesAndIssues/330), and number of citations, number of downloads, and number of shares (as indicated on the *European Radiology* website (https://link.springer.com/journal/volumesAndIssues/330) on the date the article was analyzed for this study).

### Statistical analysis

Statistical analyses were performed by using IBM SPSS Statistics for Windows (Version 20.0, IBM Corporation). Differences in dichotomous variables between open access and subscription access articles were assessed using the Pearson *χ*^2^ test. However, when the number of articles was ≤ five in one cell, Fisher’s exact test was used instead of the Pearson *χ*^2^ test. Differences in continuous variables were assessed using the Mann-Whitney *U* test. Adjustment for multiple testing was done using false positive rate control [9]. Multivariable linear regression analysis was performed to determine the association between open access and citation rate, adjusted for continent of origin of the corresponding author, subspeciality, study findings in the article title, number of authors, number of references, length of the article in pages, and number of days that the article has been online. The enter method was used for regression analysis, i.e., all independent variables were entered in a single step. Based on eight variables, approximately 500 articles were needed to be included to detect a small to medium effect size (*f*^2^ of 0.03) with a statistical power of 80% (alpha = 0.05) [10]. We also determined the association between open access and number of downloads and shares. Because articles from Europe and North America may have the benefit from some additional reputation, we also performed separate analyses for articles from these continents. *P* values less than 0.05 were considered statistically significant.

## Results

A total of 500 original studies, of which 86 (17.2%) were open access and 414 were subscription access articles (82.8%), were included. Main characteristics of these studies are displayed in Table 1. Articles with corresponding authors from Europe or North America were significantly more frequently published open access than subscription access (66.3% vs. 52.8%, *p* = 0.024; and 20.9% vs. 8.6%, *p* = 0.001, respectively). Articles with corresponding authors from Asia were significantly less frequently published open access than subscription access (12.8% vs. 37.4%, *p* < 0.001). Open access articles were significantly more frequently cited than subscription access articles (mean number of citations 12.4 vs. 8.5, *p* = 0.011). Other metrics (subspeciality, mentioning of study findings in title, number of authors, number of references, length of article in pages, and number of days online) were not significantly different between open access and subscription access articles. Results of multivariable linear regression analyses are displayed in Table 2. Open access articles were significantly more frequently cited than subscription access articles (beta coefficient = 3.588, 95% confidence interval [CI] 0.668 to 6.508, *p* = 0.016). These results remained significant when we limited our analysis to articles from North American and Europe only (beta coefficient = 4.086, 95% CI 0.418 to 7.754, *p* = 0.029). Open access articles were also significantly more frequently downloaded (beta coefficient = 759.801, 95% CI 630.917 to 888.685, *p* < 0.001) and shared (beta coefficient = 0.748, 95% CI 0.124 to 1.372, *p* = 0.019) than subscription access articles.

**Table 1:** Main characteristics of articles included

	Open access, *n* = 86	Subscription access, *n* = 414	*p* value
Continent of origin of the corresponding author (number and %)			
Asia	11 (12.8%)	155 (37.4%)	< *0.001*
Australia	0 (0%)	3 (0.7%)	0.999
Europe	57 (66.3%)	219 (52.8%)	*0.024*
North America	18 (20.9%)	35 (8.6%)	*0.001*
South America	0 (0%)	2 (0.5%)	0.999
Subspeciality (number and %)			
Breast	9 (10.5%)	30 (7.2%)	0.311
Cardiac	5 (5.8%)	27 (6.5%)	0.999
Chest	2 (2.3%)	36 (8.7%)	0.044*
Computed tomography	6 (7.0%)	36 (8.7%)	0.676
Computer applications	0 (0%)	3 (0.7%)	0.999
Contrast media	1 (1.2%)	5 (1.2%)	0.999
Education	0 (0%)	1 (0.2%)	0.999
Emergency	1 (1.1%)	2 (0.5%)	0.433
Experimental	0 (0%)	4 (1.0%)	0.999
Forensic medicine	0 (0%)	1 (0.2%)	0.999
Gastrointestinal-abdominal	7 (8.1%)	36 (8.7%)	0.999
Head-neck	0 (0%)	19 (4.6%)	0.056
Health economy	1 (1.2%)	2 (0.5%)	0.433
Magnetic resonance	9 (10.5%)	50 (12.1%)	0.719
Molecular imaging	3 (3.5%)	4 (1.0%)	0.102
Musculoskeletal	8 (9.3%)	18 (4.3%)	0.066
Neuroradiology	11 (12.8%)	38 (9.2%)	0.319
Nuclear medicine	2 (2.3%)	5 (1.2%)	0.345
Oncology	3 (3.5%)	17 (4.1%)	0.999
Pediatric	5 (5.8%)	10 (2.4%)	0.153
Physics	0 (0%)	2 (0.5%)	0.999
Ultrasound	1 (1.2%)	10 (2.4%)	0.699
Urogenital	2 (2.3%)	17 (4.1%)	0.755
Vascular-interventional	10 (11.6%)	41 (9.9%)	0.695
Mentioning of study findings in title	14 (16.3%)	66 (15.9%)	0.999
Number of authors (range)	8.6 (3–15)	8.4 (3–37)	0.136
Number of references (range)	31.3 (11–69)	30.7 (6–72)	0.810
Length of article in pages (range)	9.0 (6–14)	8.9 (4–14)	0.948
Mean number of days online (range)	1326 (1130 to 1547)	1340 (1039 to 1548)	0.302
Mean number of citations (range)	13.7 (0 to 145)	10.0 (0 to 111)	*0.021*
Mean number of downloads (range)	1244 (100–9600)	499 (0–3300)	*< 0.001*
Mean number of shares (range)	1.6 (0–26)	0.7 (0–20)	*0.035*

*p* values less than 0.05 were considered statistically significant

*Significant lost after adjustment for multiple testing using false positive rate

**Table 2:** Multivariate linear regression analysis on the association between open access publishing and number of citations, downloads, and shares. Displayed are B coefficients with 95% confidence interval between brackets and *p* value. Adjustment was also performed for continent of origin of the corresponding author and subspeciality

	Number of citations	Number of downloads	Number of shares
Open access	3.588 (0.668 to 6.508); *p = 0.016*	759.801 (630.917 to 888.685); *p < 0.001*	0.748 (0.124 to 1.372); *p = 0.019*
Study findings in the article title	1.747 (− 1.235 to 4.728); *p* = 0.250	− 24.017 (− 1.55.609 to 107.575); *p* = 0.720	0.036 (− 0.601 to 6.73); *p* = 0.911
Number of authors	0.473 (0.143 to 0.803); *p = 0.005*	5.698 (− 8.873 to 20.268); *p* = 0.443	− 0.011 (− 0.082 to 0.059); *p* = 0.756
Number of references	0.052 (− 0.057 to 0.161); *p* = 0.351	− 1.727 (− 6.556 to 3.102); *p* = 0.483	− 0.002 (− 0.026 to 0.021); *p* = 0.837
Length of the article in pages	0.136 (− 0.607 to 0.879); *p* = 0.719	3.345 (− 29.456 to 36.146); *p* = 0.841	− 0.060 (− 0.219 to 0.099); *p* = 0.457
Number of days online	0.011 (0.001 to 0.021); *p = 0.030*	0.413 (− 0.34 to 0.860); *p* = 0.070	0.001 (− 0.001 to 0.003); *p* = 0.544

*P* values less than 0.05 were considered statistically significant

## Discussion

The results of our study show that open access articles in *European Radiology* are significantly and independently more frequently cited than subscription access articles. This can be explained by the facts that open access by definition does not require a journal subscription or payment of a fee to read the article, open access offers potentially faster and easier article access even to subscribers because there is no need to login, and open access articles are also published in PubMed Central, which improves article visibility. Altogether, this may increase the number of article reads and subsequent citations. Interestingly, articles from Europe and North America were significantly more frequently published open access. The opposite was true for articles with corresponding authors from Asia. It can be speculated that there are more European and North American institutions who have an agreement with the publisher to cover open access charges or there may be more study funders from Europe and North America who require open access publishing. Indeed, *Springer*, the publisher of *European Radiology*, has open access agreements with many institutions from Europe but none from Asia (https://www.springer.com/gp/open-access/springer-open-choice/springer-compact).

The author’s h-index and journal’s impact factor (which are based on citation rates) are still the most popular measures of research influence [11, 12] and they are frequently used to select candidates for positions, promotions, assignment of research grants, and even financial rewards [13, 14]. However, one may argue whether the number of citations an article receives is a good parameter to determine its scientific impact. Citation rate may rather be a measure of utility rather than of quality, because articles are not always cited because of their scientific merit. For instance, if an article has a flaw, other studies may just cite that article to point out its shortcoming. The number of times an article has been viewed or downloaded may provide a better reflection of article usage and dissemination of knowledge. The results of our study also show that open access articles in *European Radiology* are significantly and independently more frequently downloaded and shared than subscription access articles. A previous study also confirmed that open access articles are among the most downloaded articles [15]. Interestingly, they showed that there is only small overlap between the most downloaded and most cited articles [15].

Our study has some potential limitations. First, our study was a retrospective, nonrandomized observational study. Hypothetically, authors may have self-selected only those articles of higher than average quality for open access publication. However, a previous study found evidence that the citation advantage of open access articles was not because of a quality bias from authors self-selecting what to make open access [16]. Furthermore, we have attempted to control for several other article parameters which are possibly associated with being more citable. Second, we only included articles from *European Radiology*. Other renowned general radiology journals, such as *Radiology and American Journal of Roentgenology*, offer a combined type of open access, i.e., immediate open access for selected articles and delayed open access for all articles after 1 year. This would impede an unconfounded analysis with a follow-up of more than year after article publication. A follow-up time ≤ 1 year may be insufficiently long for published articles to cumulate citations. *European Radiology* only has one open access option at present, i.e., immediate open access for selected articles. This allowed us to perform a fair comparison with a mean follow-up of 1326 days for open access articles and 1340 days for subscription access articles. Third, our results only apply to original articles. We chose not to include review articles (including guidelines and systematic reviews/meta-analysis and guidelines), because these are more often highly cited and may be more often published open access. Moreover, original articles are the core of scientific research and the primary focus of most journals.

In conclusion, open access publishing is independently associated with an increased citation, download, and share rate in the field of radiology.

## Abbreviations

CI: Confidence interval
